# Relationship Between Anxiety, Depression, and Susceptibility to Severe Acute Respiratory Syndrome Coronavirus 2 Infection: Proof of Concept

**DOI:** 10.1093/infdis/jiac006

**Published:** 2022-01-12

**Authors:** Kavita Vedhara, Kieran Ayling, Ru Jia, Lucy Fairclough, Joanne R Morling, Jonathan K Ball, Holly Knight, Holly Blake, Jessica Corner, Chris Denning, Kirsty Bolton, Hannah Jackson, Carol Coupland, Patrick Tighe

**Affiliations:** School of Medicine, University of Nottingham, Nottingham, United Kingdom; School of Medicine, University of Nottingham, Nottingham, United Kingdom; School of Medicine, University of Nottingham, Nottingham, United Kingdom; School of Life Sciences, University of Nottingham, Nottingham, United Kingdom; School of Medicine, University of Nottingham, Nottingham, United Kingdom; NIHR Nottingham Biomedical Research Centre, University of Nottingham, Nottingham, United Kingdom; School of Life Sciences, University of Nottingham, Nottingham, United Kingdom; Biodiscovery Institute, University of Nottingham, Nottingham, United Kingdom; School of Medicine, University of Nottingham, Nottingham, United Kingdom; NIHR Nottingham Biomedical Research Centre, University of Nottingham, Nottingham, United Kingdom; School of Health Sciences, University of Nottingham, Nottingham, United Kingdom; University Executive Board, University of Nottingham, Nottingham, United Kingdom; Biodiscovery Institute, University of Nottingham, Nottingham, United Kingdom; School of Mathematical Sciences, University of Nottingham, Nottingham, United Kingdom; School of Life Sciences, University of Nottingham, Nottingham, United Kingdom; School of Medicine, University of Nottingham, Nottingham, United Kingdom; School of Life Sciences, University of Nottingham, Nottingham, United Kingdom

**Keywords:** antibodies, anxiety, COVID-19, depression, SARS-CoV-2

## Abstract

**Background:**

Psychological factors can influence susceptibility to viral infections. We examined whether such influences are evident in severe acute respiratory syndrome coronavirus 2 (SARS-CoV-2) infection.

**Methods:**

Participants (n = 102) completed measures of anxiety, depression, positive mood, and loneliness and provided a blood sample for the measurement of antibodies to the SARS-CoV-2 spike and nucleocapsid proteins.

**Results:**

SARS-CoV-2 was significantly negatively associated with anxiety and depression. The model remained significant after adjustment for age and gender, although anxiety and depression were no longer significant independent predictors.

**Conclusions:**

These findings offer early support for the hypothesis that psychological factors may influence susceptibility to SARS-CoV-2 infection.

## Teaser

Risk of SARS-CoV-2 seropositivity is associated negatively with anxiety and positively with depression. The least anxious may be more likely to engage in behaviors that increase risk of exposure and infection, whereas depression may be secondary to the original infection.

Severe acute respiratory syndrome coronavirus 2 (SARS-CoV-2), the virus that causes coronavirus 2019 (COVID-19) infection, has proven to be a pernicious adversary. At the time of writing, the virus has resulted in an estimated 150 million infections and over 3 million deaths worldwide. However, what factors determine susceptibility to the infection? Previous research with other respiratory viruses suggests psychological and social factors may also influence susceptibility to the infection [1]. Viral challenge studies in particular have shown that the greatest risk of disease occurs in individuals contending with chronic stressors (of 1 month or longer in duration) and where the sources of stress are interpersonal or employment related [2]. In comparison, the experience of positive emotions and social support confer protection against viral infection [2].

This previous work suggests that several of the psychological and social consequences of the SARS-CoV-2 pandemic (ie, chronic financial and interpersonal stressors, increased negative and reduced positive emotions, and social isolation) may also have implications for an individual’s risk of contracting SARS-CoV-2. To examine this hypothesis, we conducted an exploratory observational study in which we investigated the relationship between COVID-19 infection (as indicated by the presence of antibodies to the SARS-CoV-2 spike and nucleocapsid proteins) and psychological factors previously shown to be associated with disease susceptibility (ie, positive mood and loneliness) and known to have been affected by the pandemic (ie, anxiety and depression [3]).

## METHODS

Eligibility criteria specified that participants should be aged 18 or over. Participants were staff and students at a higher education institution in the United Kingdom participating in the institution’s COVID-19 surveillance program. Participants were recruited through word of mouth and a campus-wide campaign and participation was voluntary. All participants were recruited between March 12, 2020 and February 17, 2021. Because the present sample was recruited opportunistically alongside the COVID-19 surveillance program and the research was exploratory and hypothesis-generating, no sample size target was set a priori.

### Procedures

As part of the COVID-19 surveillance program, participants who agreed to antibody testing received a finger-prick blood sample kit including a disposable lance, a barcoded 10-µL Mitre sampling stick (Neoteryx), and sampling instructions. Dried blood samples were assayed for immunoglobulin (Ig)G antibodies to SARS-CoV-2 spike and nucleocapsid proteins using enzyme-linked immunosorbent assay, and all assays were performed on Opentrons OT-2 Precision liquid handling robots. Methods have been reported previously [4]. Seropositivity was indicated according to the ratio method, ie, ratio of the average sample optical density (OD)/average negative OD. According to this approach, a ratio of ≥1.3 = seropositive, a ratio between 1.1 and <1.3 = indeterminate, and a ratio <1.1 = seronegative.

Participants also completed an online survey (https://www.onlinesurveys.ac.uk/) that captured the following: demographic information (age, gender, ethnicity); and COVID-19 testing history and psychological constructs previously associated with an individual’s risk of contracting a viral infection, ie, depression (Patient Health Questionnaire [5] [PHQ], α = .89), anxiety (Generalized Anxiety Disorder Scale [6] [GAD], α = .91), positive mood (Scale of Positive and Negative Experience [7] [SPANE], α = .95), and loneliness (single item: “How often have you felt lonely in the last 2 weeks”). For all measures, participants were asked to recall their experiences over the previous 2 weeks. Surveys were completed immediately after blood samples were collected, at the point that participants registered their blood sample. Thus, the 2-week period in the surveys included the point of blood sample provision.

### Statistical Analysis

All analyses were performed using SPSS (version 25). Summary statistics were calculated for the total group of participants and split by seropositivity status using counts and percentages and means (with standard deviations) or medians (interquartile range) as appropriate. Anxiety and depression scores were also dichotomized according to established thresholds for diagnosis of “caseness” (PHQ-9 score greater than or equal to 10, GAD-7 score greater than or equal to 8) [8]. Seven individuals were categorized as having an “indeterminant” antibody result and were excluded from all analyses. The associations between psychological factors (anxiety, depression, positive mood, and loneliness) and the binary SARS-CoV-2 seropositivity outcome were examined using logistic regression controlling for age and gender. These latter indices were included in view of evidence that the risk of infection may be greater in males and older individuals [9, 10]. Study analyses were not preregistered.

### Patient and Public Involvement

We convened a virtual Patient and Public Involvement Group to support this research. Individuals participated via Microsoft Teams in one-to-one or group discussions that informed the length and structure of the survey (eg, order of presentation of items) and language of the information sheet (ie, to maximize interest in the research).

### Ethical Approval

Ethical approval was granted from the University of Nottingham Faculty of Medicine and Health Sciences Ethics Committee (reference number 96-0920).

### Patient Consent Statement

All participants received a participant information sheet outlining the nature of the research and completed an online consent form.

## RESULTS

### Cohort Characteristics

Severe acute respiratory syndrome coronavirus 2 antibody status and survey data were available for N = 102 individuals. Of these, N = 85 (83%) were seronegative and N = 17 (17%) were seropositive (as indicated by serum levels of IgG antibody to SARS-CoV-2 spike and nucleocapsid combined). Of participants who reported a previous positive COVID-19 test result (N = 7), the average number of days since their test was 74 days (range, 51–105 days). A summary of the characteristics of the whole cohort and according to seropositivity status is shown in Table 1.

**Table 1.** Cohort Characteristics

Characteristics	All Participants (n = 102)	Seronegative (n = 85)	Seropositive (n = 17)
Age (mean, SD)	33.13 (13.80)	34.62 (14.01)	25.65 (10.05)
Gender (n, %)			
Male	36 (35.3%)	31 (36.5%)	5 (29.4%)
Female	66 (64.7%)	54 (63.5%)	12 (70.6%)
Previous positive COVID-19 test (yes)	7 (14.6%)	2 (2.4%)	5 (29.4%)
Ethnicity (n, %)			
White—British, Irish, other	89 (87.3%)	74 (87.1%)	15 (88.2%)
Other ethnic group	11 (10.8%)	9 (10.6%)	2 (11.8%)
Prefer not to say	2 (2.0%)	2 (2.4%)	0
Psychological Outcomes			
Positive mood (mean, SD)	20.55 (4.93)	20.46 (5.00)	21.00 (4.73)
Loneliness (mean, SD)	2.33 (1.32)	2.26 (1.36)	2.71 (1.05)
Depression (median, IQR)	5.00 (1.00–8.75)	4.00 (1.00–8.50)	6.00 (5.00–10.00)
Anxiety (median, IQR)	3.00 (0.00–6.00)	3.00 (0.00–6.00)	3.00 (1.50–6.00)
Depression cases (n, %)	85 (83.3%)	17 (20%)	5 (29.4%)
Anxiety cases (n, %)	17 (16.7%)	14 (16.5%)	2 (11.8%)

Abbreviations: IQR, interquartile range; SD, standard deviation.

### Psychological Factors Associated With Severe Acute Respiratory Syndrome Coronavirus 2 Seropositivity

The results from the first step of the multivariable logistic regression model suggested that the likelihood of being seropositive was significantly associated with anxiety and depression: with the likelihood being 23% lower per unit increase in anxiety (odds ratio [OR] = 0.77; 95% confidence interval [CI], .60–.98), and 27% higher per unit increase in depression (OR = 1.27; 95% CI, 1.05–1.54). After adjusting for age and gender, anxiety and depression were no longer significant independent predictors of seropositivity. Although the overall model remained statistically significant, the direction of the relationships remained unchanged and the effect on ORs was modest (see Table 2).

**Table 2.** Multivariable Logistic Regression Model Showing Associations Between Demographic and Psychological Variables and SARS-CoV-2 Antibody Status

Predictors	Odds Ratio	95% CI Lower	95% CI Upper	*β*	*p*
**SARS-CoV-2 antibody status**					
**Step 1**					
Depression	1.27	1.05	1.54	0.24	.02∗
Anxiety	0.77	0.60	0.98	-0.26	.03∗
Positive mood	1.13	0.97	1.33	0.13	.13
Loneliness	1.46	0.87	2.46	0.38	.15
**Step 2**					
Age (per year)	0.96	0.90	1.02	-0.05	.14
Male	0.77	0.21	2.88	-0.26	.70
Depression	1.18	0.96	1.45	0.17	.11
Anxiety	0.79	0.62	1.00	-0.24	.05
Positive mood	1.08	0.91	1.28	0.72	.41
Loneliness	1.34	0.78	2.31	0.29	.30

*p*<.05

Step 1: Nagelkerke R^2^=0.16, n=102; Model: χ^2^(4)=10.18, *p=*.038

Step 2: Nagelkerke R^2^=0.20, n=102; ; Model: χ^2^(6)=12.86, *p=*.045

## DISCUSSION

To our knowledge, this is the first reported analysis of the relationship between psychological factors and SARS-CoV-2 seropositivity. Our findings suggest that psychological factors may be related to the likelihood of SARS-CoV-2 infection, with our unadjusted analyses suggesting that lower levels of anxiety, and higher levels of depression, are significantly and independently associated with SARS-CoV-2 seropositivity. Several issues are worthy of further discussion.

First, our approach was predicated on antibody seropositivity being an established surrogate indicator for recent infection with SARS-CoV-2, ie, it is unlikely that someone would be seropositive for SARS-CoV-2 and have not previously been infected with the virus. Our primary analysis is consistent with this assumption. However, it is important to acknowledge several factors that can confound this relationship. First, antibodies are less likely to be detectable several months after natural infection [11]. This means that the absence of antibodies cannot be assumed to mean the absence of previous infection. Second, asymptomatic SARS-CoV-2 infection is reasonably prevalent (estimated to occur in one third of infected individuals). Such individuals may be less likely to seek SARS-CoV-2 tests (thus the absence of a previous positive COVID-19 test may not accurately represent presence/absence of previous infection). Likewise, such individuals may also be less likely to seek SARS-CoV-2 antibody testing (and may therefore be underrepresented in the present cohort). Thus, further work is needed to examine the relationship between psychological risk factors and SARS-CoV-2 infection and would ideally be examined in large cohorts undertaking asymptomatic testing for COVID-19 to mitigate the confounding effects of asymptomatic infection and antibody decay over time.

A second issue concerns the direction of the relationship between anxiety and seropositivity. This initially seems to be counterintuitive and contrary to previous work that has more commonly shown that negative moods are positively associated with risk of infection. This more expected relationship relates to negative emotions altering biological or behavioral pathways that lead to immune dysregulation and, in turn, increased disease risk [2]. We hypothesize that the existence of an inverse relationship may point to a behavioral pathway in which individuals who are less anxious may be more likely to engage in behaviors that increase their risk of exposure and infection. Some support for such a pathway can be seen in work that has shown that increased anxiety is related to a greater fear of contracting COVID-19 infection [12] and increased willingness to engage in preventative behaviors [13]. Thus, it is plausible that lower anxiety and reduced fear of infection, precipitate more risk-taking behaviors (eg, increased social contact, alcohol consumption) [14], or indeed less protective behaviors (eg, less social distancing) [13]. In post hoc analyses, we sought to interrogate this possibility by examining whether and how the relationship between anxiety, depression, and seropositivity changed when loneliness was excluded from the models (Supplementary Table 1). The models remained unchanged, although it should be noted that our measure of loneliness might be a poor proxy for social contact.

A third issue concerns the fact that individuals seropositive for SARS CoV-2 tended to also have higher depression scores. The presence of antibody indicates recent infection, with antibody levels usually detectable within a few weeks of infection and persisting for between 2 and 8 months [11], depending on the nature (ie, symptomatic versus asymptomatic) and duration of infection. This raises the possibility that the increased depressive symptoms seen in our seropositive individuals may be secondary to the original infection [15] and is consistent with evidence that depression is common post-COVID-19 infection [11].

Finally, in further post hoc analyses, we included an interaction term to capture the interaction between anxiety and depression and explore its relationship to seropositivity (Supplementary Table 2). However, the interaction was found to be nonsignificant.

## CONCLUSIONS

In conclusion, we acknowledge some potential methodological limitations. First, both the sample size and infection rate reported here are modest. This means that only relatively large effect sizes could have been reliably detected as being statistically significant. Therefore, it is important that these results are seen as hypothesis-generating and requiring replication. Second, our study design does not allow us to elucidate the temporal relationship between our psychological risk factors and SARS-CoV-2 infection. All scales asked respondents to indicate their experiences in the previous 2 weeks, and although antibody seropositivity indicates recent infection, we cannot be certain about when the infection occurred. Thus, we are, at best, describing cross-sectional relationships from which the causal direction cannot be determined.

Notwithstanding these considerations, these findings suggest that psychological factors may play a role in determining SARS-CoV-2 infection. Specifically, we have observed an inverse relationship between anxiety and SARS-CoV-2 seropositivity, which we hypothesize may be indicative of a behavioral pathway in which the least anxious individuals increase their risk of infection through engaging in more risk-taking behaviors or less engagement with protective behaviors.

## Supplementary Data

Supplementary materials are available at *The Journal of Infectious Diseases* online. Consisting of data provided by the authors to benefit the reader, the posted materials are not copyedited and are the sole responsibility of the authors, so questions or comments should be addressed to the corresponding author.

jiac006_suppl_Supplementary_Material

## Notes

***Author contributions.*** K. V., K. A., C. C., and R. J. contributed to data analysis. All authors meet the criteria for authorship as follows: design of study and contributing to drafts of the manuscript and review of final manuscript.

***Disclaimer.*** The study sponsor and funders did not play a role in the study design, collection; analysis, and interpretation of data; in the writing of the report; or in the decision to submit the paper for publication. All authors were independent of the funding source and had full access to all the data and can take responsibility for the integrity of the data and accuracy of the analyses. The views expressed are those of the author(s) and not necessarily those of the National Institute for Health Research (NIHR), the *National Health Service,* or the Department of Health.

***Financial support.*** This work described in this manuscript was funded by the University of Nottingham as part of its COVID-19 surveillance program. K. A. was supported by funding from the National Institute for Health Research School for Primary Care Research.

***Potential conflicts of interest*.** All authors: No reported conflicts of interest. All authors have submitted the ICMJE Form for Disclosure of Potential Conflicts of Interest.
