# Supplementary material for: Relationship Between Anxiety, Depression, and Susceptibility to Severe Acute Respiratory Syndrome Coronavirus 2 Infection: Proof of Concept
Source: J Infect Dis. 2022 Jan 12;225(12):2137–41. doi: 10.1093/infdis/jiac006 (PMC8807218; doi:10.1093/infdis/jiac006)
Supplement: jiac006_suppl_Supplementary_Material [file jiac006_suppl_supplementary_material.docx]

**Supplementary material**

**Supplementary Table 1:**

**Examination of effect of excluding loneliness on relationship between anxiety, depression and SARS-CoV-2 seropositivity**

|  | **Odds Ratio** | **95% CI Lower** | **95% CI Upper** | ***p*** |
| --- | --- | --- | --- | --- |
| **SARS-CoV-2 antibody status** |  |  |  |  |
| **Step 1** |  |  |  |  |
| Depression | 1.30 | 1.07 | 1.56 | .007** |
| Anxiety | 0.78 | 0.61 | 0.997 | .047* |
| Positive mood | 1.10 | 0.95 | 1.28 | .21 |
| **Nagelkerke R^2^=0.13, n=102; Model: χ^2^(3)=8.09, *p=*.044** | | |  |  |
| **Step 2** |  |  |  |  |
| Age (per year) | 0.95 | 0.90 | 1.01 | .09 |
| Male | 1.03 | 0.31 | 3.39 | .96 |
| Depression | 1.20 | 0.98 | 1.47 | .07 |
| Anxiety | 0.81 | 0.64 | 1.02 | .08 |
| Positive mood | 1.05 | 0.90 | 1.24 | .54 |
| **Nagelkerke R^2^=0.18, n=102; Model: χ^2^(5)=11.69, *p=*.039** | | | | |

**Supplementary Table 2:**

**Examination of effect of interaction between anxiety and depression and SARS-CoV-2 seropositivity**

|  | **Odds Ratio** | **95% CI Lower** | **95% CI Upper** | ***p*** |
| --- | --- | --- | --- | --- |
| **SARS-CoV-2 antibody status** |  |  |  |  |
| **Step 1** |  |  |  |  |
| Depression | 1.34 | 1.07 | 1.67 | .01* |
| Anxiety | 0.88 | 0.63 | 1.23 | .45 |
| Positive mood | 1.15 | 0.97 | 1.35 | .10 |
| Loneliness | 1.46 | 0.87 | 2.46 | .15 |
| Depression*Anxiety | 0.99 | 0.97 | 1.01 | .32 |
| **Nagelkerke R^2^=0.18, n=102; Model: χ^2^(5)=11.30, *p=*.046** | | |  |  |
| **Step 2** |  |  |  |  |
| Age (per year) | 0.96 | 0.90 | 1.02 | .19 |
| Male | 1.13 | 0.32 | 3.94 | .85 |
| Depression | 1.24 | 0.98 | 1.57 | .08 |
| Anxiety | 0.86 | 0.62 | 1.21 | .39 |
| Positive mood | 1.09 | 0.91 | 1.30 | .35 |
| Loneliness | 1.35 | 0.78 | 2.34 | .28 |
| Depression*Anxiety | 0.99 | 0.97 | 1.01 | .49 |
| **Nagelkerke R^2^=0.21, n=102; Model: χ^2^(7)=13.28, *p=*.066** | | | | |
